# Impact of targeted nursing care and nutritional support on clinical outcomes in diabetic nephropathy patients undergoing maintenance hemodialysis

**DOI:** 10.1590/1414-431X2025e14772

**Published:** 2025-11-14

**Authors:** Xiufeng Yu, Yaling Li, Xiaoyan Zhang

**Affiliations:** 1 Blood Purification Center, The Second People's Hospital of Meishan, Meishan, Sichuan, China; 2 Department of Nursing, The Second People's Hospital of Meishan, Meishan, Sichuan, China; 3 Department of Nephrology, The Second People's Hospital of Meishan, Meishan, Sichuan, China

**Keywords:** Diabetic nephropathy, Hemodialysis, Targeted nursing, Nutritional support, Intervention effect, Inflammatory indicator

## Abstract

This study aimed to investigate the impact of targeted nursing care combined with nutritional support on the clinical outcomes of diabetic nephropathy (DN) patients undergoing maintenance hemodialysis (HD). Clinical indicators such as serum creatinine (SCr), blood urea nitrogen (BUN), fasting blood glucose (FBG), hemoglobin A1c (HbA1c), and K (urea clearance) × t (dialysis time) / V (volume of urea distribution) (Kt/V), as well as inflammatory indicators such as high-sensitivity C-reactive protein (hs-CRP), interleukin (IL)-6, and tumor necrosis factor-α (TNF-α), and nutritional indicators such as transferrin (TRF), albumin (ALB), and prealbumin (PA) were measured. SF-36 quality of life scale scores were assessed, and adverse events and patient satisfaction with care were recorded. Post-intervention, the experimental group exhibited lower SCr, BUN, FBG, HbA1c, hs-CRP, IL-6, and TNF-α, and higher body mass index, Kt/V, TRF, ALB, and PA than the control group (all P<0.05). Additionally, the experimental group demonstrated higher nursing satisfaction scores, and lower total incidence of adverse events compared to the control group (all P<0.05). Targeted nursing care combined with nutritional support applied to DN patients during HD helped improve residual renal function, reduce the body's inflammatory response, improve nutritional status and the quality of life, reduce adverse events, and at the same time, improve nursing satisfaction.

## Introduction

Diabetic nephropathy (DN), a frequent and serious consequence of diabetes mellitus (DM), stands as the primary global cause of chronic kidney disease (1). This condition is marked by the buildup of extracellular matrix in the glomerular and tubulointerstitial areas, along with the thickening and hyalinization of blood vessels within the kidney (2). In about a third of diabetic patients, DN emerges following latency phases (3). Despite effective management of blood glucose and pressure, DN remains unstoppable and progresses to end-stage renal disease, requiring either dialysis or kidney transplantation (4).

Patients receiving ongoing dialysis often face the risk of protein-energy depletion, a common issue in long-term dialysis patients, leading to negative health consequences and elevated morbidity/mortality rates (5). These include accelerated loss of residual renal function, myocardial and cerebral ischemia induced by hemodialysis (HD), etc. (6). Residual renal function, which refers to the portion of the kidneys that continues to function during dialysis, plays a crucial role in controlling fluid and electrolyte balance, reducing dialysis-related complications, improving quality of life, and enhancing survival rates (7). Therefore, preserving residual renal function has become a key objective in the treatment of patients with kidney diseases (7). Dialysis patients are advised to consume 1.2 times more protein than the average population due to reduced digestive efficiency and energy depletion from dialysis (8). Consequently, nutritional assistance, such as enteral tube feeding or parenteral nutrition, ought to be contemplated as a feasible method for enhancing nutritional conditions (9). In extreme instances, HD can cause organ damage or blindness, potentially triggering adverse emotions, thereby diminishing patients' trust in the treatment. Consequently, enhanced dialogue and prompt psychological advice to patients play a role in elevating their mental well-being and eradicating adverse emotions, thus guaranteeing the efficacy of the treatment (10).

Extensive clinical studies have highlighted the significance of proactive and efficient nursing practices for DN patients receiving HD (11,12). For instance, targeted nursing care can rapidly alleviate swelling and pain in patients with limb fractures and improve their emotional states, thereby promoting health recovery and effectively enhancing patient satisfaction (13). Given that targeted nursing helps in patient recuperation and establishes a solid groundwork for patient rehabilitation, this study aimed to merge targeted nursing and nutritional support in DN patients receiving HD, followed by evaluating the clinical impact of this method on DN.

## Material and Methods

### Ethics statement

The study was approved by the Ethics Committee of The Second People's Hospital of Meishan (approval number: 20241203), and all patients signed an informed consent form.

### Participants

This study recruited 130 patients with DN undergoing hemodialysis treatment admitted to The Second People's Hospital of Meishan from April 2016 to April 2021.

#### Inclusion criteria

Patients meeting the relevant criteria (14) and diagnosed in the end stage of DN by clinical examination, undergoing regular HD for six months or more, in stable condition in the six months prior to enrollment, agreeing to participate in the study, and having no previous psychiatric diseases and cognitive disorders were included.

#### Exclusion criteria

Patients with combined acute and chronic infectious diseases, acute kidney injury, cardiac insufficiency, active bleeding, tumors within the last 1 year, accompanied by cognitive disorders or psychiatric disorders were excluded.

### Randomization process

After indicating their willingness to participate, individuals were asked to sign an informed consent form. Eligible participants were randomly allocated to either the experimental group or the control group at a 1:1 ratio using a sequential numbering system. The group assignment codes were concealed within sealed, carbon-free paper envelopes to preserve allocation blinding. These envelopes were safeguarded by a study administrator who had no direct involvement in participant recruitment, enrollment, or follow-up assessments. On the day of study inclusion, the administrator opened the sealed envelope corresponding to the participant's recruitment sequence and disclosed the group allocation number. This protocol ensured that all personnel involved in outcome assessment and data analysis remained blinded to group assignments until the study's conclusion.

### Intervention methods

Conventional therapies were preferred in all patients to control glucose and blood pressure, correct anemia, and regulate calcium and phosphorus metabolism. Patients were treated with HD three times a week, with a treatment time of 4 h/time.

#### Control group

Specialized nurses were responsible for the routine HD care, including strict glycemic control during hemodialysis sessions, administering appropriate glucose solutions to prevent hypoglycemia, closely monitoring vital signs such as body weight and blood pressure, reinforcing oral care, and providing routine health education to patients and their families.

#### Experimental group

The same routine HD care as the control group, combined with nutritional support was given for intervention. 1) An intervention team, consisting of nephrologists, HD nurses, dietitians, and psychologists, was established. The team members were regularly trained and assessed on relevant knowledge to ensure that group members were proficient in clinical practice. 2) Targeted psychological care intervention: psychological evaluation was conducted on patients upon admission to assess their psychological condition. Weekly health lectures were conducted employing straightforward language and practical scenarios to underscore the significance of a positive psychological state in enhancing treatment effectiveness and consequently, increasing the patient's therapeutic motivation. Encouraging active communication and employing suggestive language were recommended strategies to prompt patients to articulate their emotions, which could then be alleviated through conversation and guidance, ultimately leading to an improvement in their psychological well-being. 3) Targeted vascular access care: the vascular access plays a crucial role in HD, particularly for diabetic patients who commonly experience vascular sclerosis, which can lead to complications such as subcutaneous hematomas, fistula formation, and infections, ultimately impairing access functionality. Consequently, monitoring vascular access becomes imperative in the treatment process. Additionally, to ensure optimal dialysis, the dialyzer is periodically rinsed with normal saline every 45 min, while simultaneously monitoring venous pressure. Following dialysis, it is imperative to ensure proper sealing of the tube. Additionally, patients were advised to administer hot compresses on the second day to mitigate the risk of vascular sclerosis, restore elasticity in the vicinity of the fistula, enhance blood vessel volume, and prevent or minimize coagulation. 4) Targeted complication care: in the event of subcutaneous hematoma, fistula, or infections, proactive measures were taken. Timely management of infections is crucial, involving local disinfection and antibiotic medication. Furthermore, the patients were provided with guidance on enhancing their nutrition and exercise regimens, as well as increasing the protein intake to enhance their resistance to infections. In the case of subcutaneous hematomas, appropriate hemostasis measures were instructed based on the individual patient's condition. Similarly, for fistulas, patients were advised to avoid limb flexion, keep the limb on the side of the internal fistula elevated, apply warmth, and engage in light activities using the affected limb to promote blood circulation and support fistula maturation. In addition, close monitoring throughout the treatment period is essential, especially for malnourished patients, who may be at increased risk of complications. Specifically, vigilance should be exercised to detect hypoglycemic reactions such as dizziness, panic, sweating, and an elevated heart rate during the dialysis procedure. In the event of hypoglycemic reactions, patients should promptly receive interventions such as candy, sugar solutions, or, if deemed necessary, intravenous administration of 50% glucose. 5) Targeted nutritional support: a thorough assessment of the patients' physical condition was conducted to gain insight into their overall health. Based on the patients' nutritional status, general dietary recommendations can be offered to those with normal nutritional levels, while tailored nutritional recipes were developed for patients experiencing malnutrition. The recipes were developed by considering patients' clinical indicators, including blood glucose, plasma protein, and dietary habits, to achieve appropriate proportions of carbohydrates, proteins, fats, and other essential nutrients. To ensure comprehensive nutritional support, accurate rehabilitation education on nutritional knowledge was effectively communicated to patients and their families through nutrition-related guidebooks and the use of WeChat messaging app. Outpatients were directed to diligently document their daily dietary intake to facilitate the evaluation of their nutritional status and identify any issues in the dietary process upon readmission to the hospital, thereby enabling targeted guidance. Compliance was enhanced through the formation of a self-assessment based on the dietary records from various phases.

### Outcome measurements

#### Clinical indicators

Three milliliters fasting venous blood was collected from patients of the two groups before and after 1 month of intervention. Serum creatinine (SCr), blood urea nitrogen (BUN), fasting blood glucose (FBG), and hemoglobin A1c (HbA1c) were measured using the Hitachi 7600 biochemical autoanalyzer and the corresponding reagent kits (manufactured by Hitachi, Japan). Additionally, the urea clearance index [K (urea clearance) × t (dialysis time) / V (volume of urea distribution) (Kt/V)] was calculated.

#### Inflammatory indicators

Three milliliters fasting venous blood was collected from patients in both groups before and 1 month after the intervention, and inflammatory factors such as high-sensitivity C-reactive protein (hs-CRP), interleukin-6 (IL-6), and tumor necrosis factor-α (TNF-α) were tested using enzyme-linked immunosorbent assay kits. All reagent kits were purchased from Sangon Biotech Co., Ltd. (China).

#### Nutritional indicators

Body mass index (BMI) was calculated in both groups before and after 1 month of intervention. Three milliliters fasting venous blood was collected to determine transferrin (TRF), albumin (ALB), and prealbumin (PA) using the immunoturbidimetric assay, microplate method, and nephelometric assay, respectively. All reagent kits were procured from Nanjing Jiancheng Bioengineering Institute (China).

#### Quality of life

Quality of life was assessed using the SF-36 scale before the intervention and 1 month after the intervention, comparing the scores (physical functioning, body pain, social functioning, and mental health), with a total of 100 points in each dimension, and the scores were positively correlated with the quality of life (15).

#### Adverse events and satisfaction

Adverse events such as hypotension, catheter folding, hypoglycemia, tube blockage, and infection were recorded. Patients' satisfaction with nursing interventions was surveyed and categorized as very satisfied, satisfied, and dissatisfied. Nursing satisfaction = (very satisfied + satisfied) / total number of cases × 100%.

### Statistical analysis

Data were statistically analyzed using SPSS 22.0 software (IBM, USA). Data normality was assessed with the Kolmogorov-Smirnov test; normally distributed data are reported as means±SD and compared by the unpaired Student's *t*-test or paired *t*-test. Categorical data are reported as numbers and percentages [n (%)] and compared by χ^2^ test. The difference was considered to be statistically significant at P<0.05.

## Results

### General information

The analysis revealed no significant difference in terms of gender, age, course of DM, course of DN, HD period, marital status, education, and residence between the experimental group and control group (P>0.05) (Table 1).

**Table 1:** Comparison of general information between the two groups.

Indicators	Control group (n=65)	Experimental group (n=65)	*t* / *χ* ^2^ value	P value
Gender			0.032	0.858
Male	38 (58.46%)	39 (60.00%)		
Female	27 (41.54%)	26 (40.00%)		
Age (years)	56.26±6.53	57.23±7.05	-0.814	0.417
Marital status			1.242	0.743
Unmarried	6 (9.23%)	5 (7.69%)		
Married	48 (73.85%)	48 (73.85%)		
Divorced or widowed	11 (16.92%)	12 (18.46%)		
Education			2.223	0.329
Primary and below	26 (40.00%)	27 (41.54%)		
Middle school	23 (35.38%)	16 (24.61%)		
College and above	16 (24.62%)	22 (33.85%)		
Residence			1.237	0.266
Towns	46 (70.77%)	40 (61.54%)		
Villages	19 (29.23%)	25 (38.46%)		
Course of DM (years)	16.60±6.73	15.17±6.01	0.385	0.701
Course of DN (years)	4.46±1.19	4.75±1.94	-1.308	0.301
Hemodialysis duration (months)	24.49±7.69	24.08±7.22	0.318	0.751

Data are reported as means and SD or number (%) and compared by the unpaired Student's *t*-test or χ^2^ test. DM: diabetes mellitus; DN: diabetic nephropathy.

### Clinical indicators

Before the intervention, the differences in SCr, BUN, Kt/V, FBG, and HbA1c values between the experimental group and control group were not statistically significant (P>0.05). After the intervention, SCr, BUN, FBG, and HbA1c values of the experimental group were lower compared to those of the control group, and Kt/V was higher in the experimental group than in the control group (P<0.05) (Table 2).

**Table 2:** Comparison of clinical indicators before and after the intervention in the two groups.

Indicators	Time	Control group (n=65)	Experimental group (n=65)	*t* value	P value
SCr (mmol/L)	Before intervention	840.37±200.53	843.76±198.29	-0.097	0.923
	After intervention	513.52±124.30	322.23±116.22	9.099	<0.001
BUN (mmol/L)	Before intervention	21.33±4.21	21.62±4.30	-0.389	0.698
	After intervention	18.26±2.49	14.33±2.16	9.612	<0.001
Kt/V	Before intervention	1.83±0.22	1.81±0.20	0.542	0.589
	After intervention	2.14±0.32	2.39±0.34	4.317	<0.001
FBG (mmol/L)	Before intervention	11.47±1.42	11.39±1.38	0.328	0.744
	After intervention	8.87±1.67	6.24±1.53	9.361	<0.001
HbA1c (%)	Before intervention	8.46±2.09	8.22±1.87	0.691	0.491
	After intervention	7.12±1.53	5.49±1.02	7.145	<0.001

Data are reported as means and SD and compared by the unpaired Student's *t*-test. SCr: serum creatinine; BUN: blood urea nitrogen; Kt/V: K (urea clearance) × t (dialysis time) / V (volume of urea distribution); FBG: fasting blood glucose; HbA1c: hemoglobin A1c.

### Inflammatory factors

Before the intervention, there were no statistically significant differences in hs-CRP, IL-6, and TNF-α between the two groups (P>0.05). After the intervention, hs-CRP, IL-6, and TNF-α levels were lower in the experimental group than in the control group (P<0.05) (Table 3).

**Table 3:** Comparison of inflammatory factors before and after the intervention in the two groups.

Indicators	Time	Control group (n=65)	Experimental group (n=65)	*t* value	P value
hs-CRP (mg/L)	Before intervention	13.87±3.35	13.56±3.09	0.548	0.585
	After intervention	8.12±1.32	5.88±0.78	11.782	<0.001
IL-6 (pg/mL)	Before intervention	133.56±14.48	135.78±16.02	-0.828	0.409
	After intervention	84.87±5.33	58.90±4.39	30.323	<0.001
TNF-α (ng/L)	Before intervention	17.54±1.79	17.89±1.88	-1.089	0.278
	After intervention	9.23±1.34	6.88±0.98	11.412	<0.001

Data are reported as means and SD and compared by the unpaired Student's *t*-test. hs-CRP: high-sensitivity C-reactive protein; IL-6: interleukin-6; TNF-α: tumor necrosis factor-α.

### Nutritional indicators

A comparison of BMI, TRF, ALB, and PA between the experimental group and control group before intervention did not reveal any significant differences (P>0.05). After intervention, BMI, TRF, ALB, and PA in the experimental group were higher than those in the control group (P<0.05) (Table 4).

**Table 4:** Comparison of nutritional indicators before and after the intervention in the two groups.

Indicators	Time	Control group (n=65)	Experimental group (n=65)	*t* value	P value
BMI (kg/m^2^)	Before intervention	18.34±1.22	18.22±1.30	0.634	0.588
	After intervention	20.12±2.20	21.87±1.70	-5.076	<0.001
TRF (g/L)	Before intervention	1.88±0.29	1.90±0.27	-0.416	0.678
	After intervention	2.23±0.22	2.52±0.30	-6.063	<0.001
ALB (g/L)	Before intervention	31.89±4.02	31.67±3.89	0.318	0.751
	After intervention	35.26±4.08	38.02±3.15	-4.316	<0.001
PA (g/L)	Before intervention	0.25±0.09	0.26±0.09	-0.595	0.553
	After intervention	0.33±0.07	0.40±0.11	-4.289	<0.001

Data are reported as means and SD and compared by the unpaired Student's *t*-test. BMI: body mass index; TRF: transferrin; ALB: albumin; PA: prealbumin.

### Quality of life

Physiological function, body pain, social function, and mental health were not statistically different between the experimental group and the control group before the intervention (P>0.05). After the intervention, physiological function, body pain, social function, and mental health scores of the experimental group were notably higher than those of the control group (P<0.05) (Table 5).

**Table 5:** Comparison of quality of life before and after the intervention in the two groups.

Indicators	Time	Control group (n=65)	Experimental group (n=65)	*t* value
Physiological function	Before intervention	26.43±4.97	26.02±4.71	0.489
	After intervention	49.26±5.95	57.69±5.20	-8.606
Body pain	Before intervention	29.85±3.26	30.29±3.35	-0.77
	After intervention	38.40±4.20	47.40±5.08	-11.007
Social function	Before intervention	46.08±4.34	45.37±4.90	0.872
	After intervention	53.86±5.24	57.23±5.92	-3.435
Mental health	Before intervention	42.28±5.33	41.89±4.98	0.425
	After intervention	56.23±4.40	63.25±5.22	-8.287

Data are reported as means and SD and compared by the unpaired Student's *t*-test.

### Adverse events and patient satisfaction

The nursing satisfaction of patients in the experimental group was higher than in the control group (P<0.05). The total incidence of adverse events in the experimental group was lower compared with that in the control group (P<0.05) (Table 6).

**Table 6:** Comparison of adverse events and patient satisfaction in the two groups.

	Control group (n=65)	Experimental group (n=65)	*χ* ^2^ value	P value
Hypotension	4 (6.15%)	2 (3.08%)	-	-
Catheter folding	3 (4.62%)	0 (0.00%)	-	-
Catheter blockage	5 (7.69%)	2 (3.08%)	-	-
Infection	2 (3.08%)	1 (1.54%)	-	-
Hypoglycemia	8 (12.31%)	3 (4.62%)	-	-
Total incidence (%)	22 (33.85%)	8 (12.31%)	8.493	0.004
Satisfaction (%)	52 (80.00%)	63 (96.92%)	9.119	0.003

Data are reported as number (%) and compared by the χ^2^ test.

## Discussion

Patients diagnosed with DN commonly present with hypoproteinemia, which subsequently results in the accumulation of fluid in the thoracic and abdominal regions. This fluid buildup exacerbates the patient's pain, requiring the implementation of dialysis to preserve renal function. The clinical management of DN patients relies heavily on HD, requiring a range of nursing strategies. As a result, routine care alone fails to adequately meet these demands, thereby highlighting the need for continuous improvement in the quality of nursing care (16). Therefore, for the treatment of DN patients receiving HD, this trial developed a targeted nursing plan along with nutritional support.

BUN and SCr are indicators of kidney function (17), and FPG and HbA1c are indicators of dysglycemia (18). In the current study, targeted nursing and nutritional support used to control DN progression achieved a better clinical outcome in the experimental group compared to the control group in terms of BUN, SCr, FPG, and HbA1c. Additionally, Kt/V is a commonly used method for measuring dialysis adequacy in clinical practice, with a higher value indicating better dialysis efficacy (19). After the intervention in this study, the Kt/V value in the experimental group was higher than that in the control group. This suggests that targeted nursing combined with nutritional support leads to more adequate and effective dialysis treatment, contributing to the maintenance of internal environmental stability in the body. DN progression often involves microinflammation as a common pathway (20). DN development has been controlled clinically using anti-inflammatory strategies targeting inflammatory mediators (21). CRP is not only an inflammatory biomarker but a pathogenic indicator in DN (22). IL-6 signaling participates in inflammation responses central to DN progression (23). TNF-α is a proinflammatory cytokine associated with renal injuries in patients with DN (24). This trial detected CRP, IL-6, and TNF-α to assess the inflammatory response in DN patients and they were lower in the experimental group than in the control group, supporting the efficacy of targeted nursing and nutritional support in controlling inflammation in DN patients.

In addition, nutritional factors such as BMI, TRF, ALB, and PA were found to be higher in the experimental group, suggesting that targeted nursing and nutritional support improved the nutritional status in DN patients. During the treatment process, a reasonable diet is an effective way to control blood glucose and blood lipids. By monitoring the patient's blood glucose, blood pressure, and daily calorie intake, appropriate recipes can be developed to provide targeted dietary guidance, which has a positive impact. In addition, the quality of life was improved in the experimental group, with higher satisfaction and a lower incidence of adverse events. This also means that for diabetic chronic renal failure patients undergoing HD, the adoption of targeted nursing care plays a certain role in reducing the incidence of complications and effectively improving nursing satisfaction. The primary rationale behind this phenomenon lies in the favorable influence of targeted care on patients. Following targeted nursing interventions, patients experience varying degrees of improvement in their psychological and physiological conditions, thereby fostering a closer nurse-patient relationship and augmenting the therapeutic outcome. In the context of clinical management for individuals with DN, targeted care improves patient compliance with treatment, thereby positively impacting patient prognosis and the evolution of the nurse-patient relationship.

In conclusion, implementing targeted nursing combined with nutritional support for patients with DN during HD is beneficial for improving residual renal function, reducing systemic inflammatory responses, enhancing nutritional status and quality of life, decreasing adverse events, and simultaneously increasing nursing satisfaction. This holds significant clinical implications. It provides a feasible and synergistic intervention protocol for clinical practice, offering new evidence for the formulation of individualized treatment strategies by optimizing nutritional plans and dialysis management.

This study also has certain limitations. Firstly, the relatively small sample size and single-center design may affect the generalizability of the findings, requiring validation through larger multicenter studies. Secondly, this study lacked long-term follow-up. In future research, long-term follow-up of patients can be conducted to investigate the long-term effects of targeted nursing combined with nutritional support on patients.

## Data Availability

All data generated or analyzed during this study are included in this published article.

## References

[1] Elendu C, Okah MJ, Fiemotongha KDJI, Adeyemo BI, Bassey BN, Omeludike EK (2023). Comprehensive advancements in the prevention and treatment of diabetic nephropathy: a narrative review. Medicine (Baltimore).

[2] Kanwar YS, Sun L, Xie P, Liu FY, Chen S (2011). A glimpse of various pathogenetic mechanisms of diabetic nephropathy. Annu Rev Pathol.

[3] Samsu N (2021). Diabetic nephropathy: challenges in pathogenesis, diagnosis, and treatment. Biomed Res Int.

[4] Fioretto P, Barzon I, Mauer M (2014). Is diabetic nephropathy reversible?. Diabetes Res Clin Pract.

[5] Sabatino A, Regolisti G, Karupaiah T, Sahathevan S, Singh BKS, Khor BH (2017). Protein-energy wasting and nutritional supplementation in patients with end-stage renal disease on hemodialysis. Clin Nutr.

[6] Selby NM, Kazmi I (2019). Peritoneal dialysis has optimal intradialytic hemodynamics and preserves residual renal function: why isn't it better than hemodialysis?. Semin Dial.

[7] Xie J, Liu X, Ling Y, Ge S, Yao Y (2025). The impact of low-protein diet on residual renal function in dialysis patients: a systematic review and metaanalysis. BMC Nephrol.

[8] Hoshino J (2021). Renal rehabilitation: exercise intervention and nutritional support in dialysis patients. Nutrients.

[9] Chan W (2021). Chronic kidney disease and nutrition support. Nutr Clin Pract.

[10] Kundu A, Dey P, Sarkar P, Karmakar S, Tae IH, Kim KS (2020). Protective effects of Croton hookeri on streptozotocin-induced diabetic nephropathy. Food Chem Toxicol.

[11] Rout P, Jialal I, Doerr C (2025). StatPearls.

[12] Yu L, Lei C, Hao J (2023). Study on the effect of high-quality nursing in patients with diabetic nephropathy. Panminerva Med.

[13] Wang X, Xu H, Wu X (2024). Effectiveness of targeted nursing measures to relieve swollen limb pain after extremity fracture. Altern Ther Health Med.

[14] National Kidney F (2012). KDOQI Clinical Practice Guideline for Diabetes and CKD: 2012 Update. American journal of kidney diseases: the official journal of the National Kidney Foundation.

[15] Li L, Wang HM, Shen Y (2003). Chinese SF-36 Health Survey: translation, cultural adaptation, validation, and normalisation. J Epidemiol Community Health.

[16] Hinoshita F, Ando R, Sakai R, Kuriyama S (2014). Hemodialysis-associated problems to solve: current and future. ScientificWorldJournal.

[17] Griffin BR, Faubel S, Edelstein CL (2019). Biomarkers of drug-induced kidney toxicity. Ther Drug Monit.

[18] Duan D, Kengne AP, Echouffo-Tcheugui JB (2021). Screening for diabetes and prediabetes. Endocrinol Metab Clin North Am.

[19] Churchill BM, Patri P (2021). The Nitty-Gritties of Kt/V(urea) calculations in hemodialysis and peritoneal dialysis. Indian J Nephrol.

[20] Wada J, Makino H (2013). Inflammation and the pathogenesis of diabetic nephropathy. Clin Sci (Lond).

[21] Rayego-Mateos S, Rodrigues-Diez RR, Fernandez-Fernandez B, Mora-Fernández C, Marchant V, Donate-Correa J (2023). Targeting inflammation to treat diabetic kidney disease: the road to 2030. Kidney Int.

[22] Tang Y, Fung E, Xu A, Lan HY (2017). C-reactive protein and ageing. Clin Exp Pharmacol Physiol.

[23] Feigerlova E, Battaglia-Hsu SF (2017). IL-6 signaling in diabetic nephropathy: From pathophysiology to therapeutic perspectives. Cytokine Growth Factor Rev.

[24] Elmarakby AA, Sullivan JC (2012). Relationship between oxidative stress and inflammatory cytokines in diabetic nephropathy. Cardiovasc Ther.

